# Analysis of E-mail Account Probing Attack Based on Graph Mining

**DOI:** 10.1038/s41598-020-63191-5

**Published:** 2020-04-29

**Authors:** Yi Wen, Xingshu Chen, Xuemei Zeng, Wei Wang

**Affiliations:** 1 College of Cybersecurity, Sichuan University, Chengdu, 610065 China; 2 Cybersecurity Research Institute, Sichuan University, Chengdu, 610065 China

**Keywords:** Computer science, Scientific data

## Abstract

E-mail has become the main carrier of spreading malicious software and been widely used for phishing, even high-level persistent threats. The e-mail accounts with high social reputation are primary targets to be attacked and utilized by attackers, suffering a lot of probing attacks for a long time. In this paper, in order to understand the probing pattern of the e-mail account attacks, we analyse the log of email account probing captured in the campus network based on graph mining. By analysing characteristics of the dataset in different dimensions, we find a kind of e-mail account probing attack and give it a new definition. Based on the analysis results, its probing pattern is figured out. From the point of probing groups and individuals, we find definitely opposite characteristics of the attack. Owing to the probing pattern and its characteristics, attacks can escape from the detection of security devices, which has a harmful effect on e-mail users and administrators. The analysis results of this paper provide support for the detection and defence of such distributed attacks.

## Introduction

E-mail has become a common social tool on the internet and its security has been a hot-spot. Attackers prefer to use e-mail to pass on malware or phishing so that they can carry out different kinds of attacks such as some traditional network attacks or the APT penetration attack. In recent years, the proportion of users attacked by malicious e-mail has increased significantly^1^. E-mail account probing is the most common early action for attackers. The main purpose of email account probing is to crack passwords and open a breakthrough for subsequent attacks. Accounts with high social reputation, especially those of users in universities and well-known enterprises, are the main targets, suffering a lot of probing attacks. Spam and some harmful attachments such as worms, Trojans and ransomware, will be sent by the compromised e-mail accounts, which may bring huge losses to organizations or individuals. The high social reputation attribute of e-mail accounts aggravates the success rate of probing attacks. In the past few years, there have been a series of attacks that successfully attacked government systems, well-known businesses, some politicians and organizations^2^. How to protect e-mail accounts is the most important task to reduce the risk of network attacks.

The security of e-mail has attracted many scholars attention because of its importance and necessity. At present, the main research of e-mail security is about identification and filtering of malicious e-mails. The commonly used technologies include detection and filtering of malicious e-mails based on expert knowledge, machine learning or automatic method of rule extraction^3–7^. However, with the development of network attack technology, attacks against e-mail have gradually evolved from traditional ones of single and isolated source to automated and distributed ones^8^. Many attackers including some APT organizations probe e-mail accounts and send spam by distributed botnets^9^. Attacks such as probing accounts in the distributed and covert way, can escape from detection of malicious e-mail. Such distributed and covert attacks are adopted by growing attackers because of good concealment and high attack efficiency. Traditional methods have some limitations in distinguishing malicious login behaviours from benign ones. Attackers can escape from detection of malicious e-mail in this way to implement intrusions into the Intranet. Nevertheless, there are few methods to detect distributed e-mail attacks in prior research, and there is also a lack of description and analysis of e-mail attackers’ behaviours.

How to detect and defend this kind of distributed probing attack, is one of the key problems for e-mail security. In order to figure out how attackers probe accounts, we need to analyse characteristics of the probing pattern, describe attack sources’ behaviours, and propose targeted solutions. However, the distributed characteristics of such attacks make it difficult to extract features directly by expert knowledge from a little amount of data. The analysis of a large number of data is needed for researchers to understand the probing pattern. Accordingly, methods of data mining are needed for analysis to figure out the hidden information of dataset. Graph mining is a subclass of data mining, which has become a popular area of research in recent years because of its numerous applications in a wide variety of practical fields^10^. As methods of graph mining can reflect characteristics and predict the evolution of data, they can provide support for the analysis and research in theory.

Theories of graph mining have attracted extensive attention in the study of human’s data about network and communication in recent years. Zhou *et al*.^11^ have summarized the research methods of spatial and temporal characteristics of human behaviours in recent years. Wang *et al*.^12^ introduce recent progress in the study of coevolution spreading dynamics. Jiang *et al*.^13^ studied the data on phone calls and three abnormal communication griioups are obtained through the analysis of data’s distribution. By analysing the distribution of Twitter data, Bovet *et al*.^14^ concluded the influence of fake news on the U.S. general election. As for time characteristics in networks, Masuda *et al*.^15^ proposed a method to assign discrete states to the systems in social temporal networks. Bai *et al*.^16^ analysed two networks’ temporal structures for the early detection of infectious disease. In the analysis of network traffic behaviours, many researchers use methods of graph mining to solve different problems. Francois *et al*.^17^ proposed using flows to construct graph to analyse network communication pattern and used Authority as well as Hub eigenvalues of graph to detect botnets. Weigert *et al*.^18^ proposed a graph-based community discovery method, which showed that the IP addresses of the community were similar on network flows and it could identify low-intensity attacks to multiple hosts. Ye^19^ proposed using Graphlet to quantify the correlation of eigenvalues, fusing the attributes of graph nodes and Graphlet attributes to describe individual behaviour characteristics on the internet. Prior research shows that analysis methods based on graph mining theories are feasible in the description of network traffic behaviours.

Accordingly, in this work, we use methods of graph mining such as temporal analysis and graph node evaluation to analyse the distribution of e-mail probing data collected in campus network. We focus on characteristics of the data in time dimension and the spatial dimension of the space which is constructed by probing sources and targets. Attackers’ behaviours and probing patterns are described from the views of individual probing sources and the whole data set. Results of our analysis can be used to assess the security risk of e-mail accounts and provide help for the security defence of colleges or enterprises’ e-mail systems. The main contributions of this paper are as follows.

First, our work is based on the real e-mail traffic data in campus network. The dataset we collected contains abundant information, which is of high research value especially for analysing attackers behaviour tendency in e-mail system.

Secondly, we describe and analyse attackers’ behaviours in the network traffic, which makes up the lack of research on such distributed e-mail probing attack.

Finally, this paper uses theories of temporal analysis, network structure and graph node evaluation as the analysis methods. We analyse the temporal feature of the dataset and construct networks to figure out the correlation between attackers and targets, which can provide a reference for such kind of analysis of security data, especially the distributed attack data which is similar to our dataset.

The structure of the rest parts of this paper is as follows. The second chapter introduces the data set of this paper. Then, the third chapter is about the analysis methods used in three aspects, including time characteristics, network model structures and network node attributes. The fourth chapter demonstrates analysis results of data set based on above methods, analysing the characteristics of attack behaviour in different dimensions. Chapter five is the summary and discussion of this paper.

## Dataset

The original dataset is the e-mail login data log collected from a campus network. It contains traffic records of failed login in 333 days. Each record includes login time, login IP address, network segment of IP address, login email username and other fields. We consider that some normal users may make a mistake entering passwords which would generate a login failed record. Therefore, we count the number of login failures for each class C network segment and filter out records with the total number of login failures less than 4 in the previous seven days. Considering the convenience of analysis, we aggregate data with one day as the minimum unit. For the purpose of clearing the sensitive information, we anonymize the data set, randomly numbering the date, IP address, network segment and username of each record. In this way, each field of a record has an ID to represent it. After the pre-processing, the campus network e-mail probing data set (hereinafter referred to as CNEPD) is formed. The basic information of CNEPD is shown in Table 1.

**Table 1:** Basic information about CNEPD.

Records	Dates	IP Address	Class C Network Segments	E-mail Accounts	Total attacks
764,537	333	101,460	1,737	102,238	3,292,831

Each record in CNEPD consists of attack date (Date), IP address (IP), class C network segment (Segment), username of e-mail account (Username) and the number of probing attacks (Count). The format of record is as follow:

$$\{Date,IP,Segment,Username,Count\}$$

Attackers frequently change their IP addresses to avoid tracking or sometimes use compromised hosts which are under the same class C network segment^20^. Accordingly, we assume that all the IP addresses are class C addresses, which indicate that if the first three digits of different IP addresses are the same, they belong to the same network segment. Therefore, we change the minimum unit of probing source from IP address to network segment.

## Methods

The analysis methods consist of two parts. First of all, we analyse the time distribution of CNEPD, so as to obtain the time characteristics of each probing source. Secondly, we establish a probing relationship network based on attacker segments and e-mail accounts. Thus, we can find out whether there is a certain relationship between probing sources and targets. Based on the established network, we analyse the relationship between probing sources by mapping the network to a new one to find out the similarity between attackers. From the time dimension and the spatial dimension of the network, we can get different characteristics which can describe the pattern of this kind of probing attack. Section 2.1 is the time feature extraction method, and sections 2.2 and 2.3 are the methods of network structure analysis. Since CNEPD has certain characteristics of distributed attacks, we consider the following two aspects to focus on behaviours: (1) overall probing behaviour characteristics of CNEPD; (2) the behaviour characteristics of single node. Analysis results from these two aspects can accurately and comprehensively reflect the characteristics of behaviour patterns to describe such probing attacks in terms of groups and individuals.

### Time feature extraction

**Figure 1:**
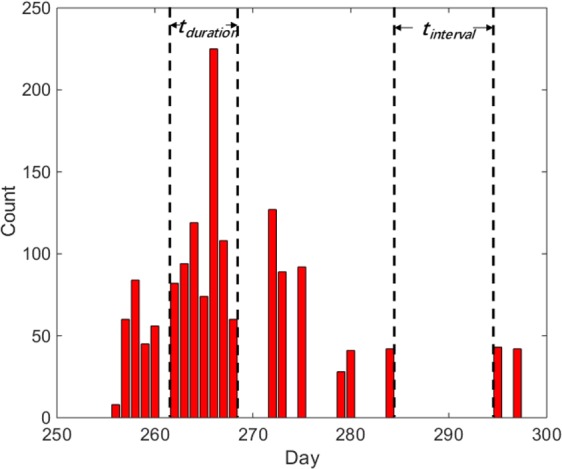
An example of *t*_*interval*_ and *t*_*duration*_. The abscissa is the date,ranging from 250 to 300, and the ordinate is the number of attacks corresponding to each date.

Time characteristics are important attributes to describe the probing pattern because they can manifest the tendency of attacks in time dimension. In the description of time characteristics of graph or complex networks, the concepts of burstiness and memory were proposed in^21^ . The burstiness is the time distribution characteristic of time interval among nodes, while the memory indicates the time when nodes continue to appear. Learning from these two characteristics above, two definitions are proposed to describe time characteristics of the e-mail probing behaviour. Figure 1 shows a part of the No.80 nodes probing distribution of dates and the number of attacks, and definitions of time characteristics are as follows:

***Definition 1: Probing Interval*** (*t*_*interval*_): Probing interval refers to the number of days between two adjacent probing attacks. As shown in Fig. 1, from day 285 to 296 there is no probing, and the date difference is the *t*_*interval*_.

***Definition 2: Probing Duration*** **(***t*_*duration*_**)**: Probing duration refers to the number of consecutive days that probing attacks continuously occur. As shown in Fig. 1, from day 261 to 269, probing attacks persisted, and the date difference is the *t*_*duration*_.

*t*_*interval*_ and *t*_*duration*_ are calculated to describe the time distribution of CNEPD, which can reflect the tendency of attackers’ probing patterns. Based on the large data analysis platform CSRI-BDP established in our laboratory, we aggregate CNEPD to extract time features according to algorithm 1. The input of the algorithm is CNEPD, the login failed data set, which is stored in the Hadoop Distributed File System (HDFS). HDFS is the storage system of Hadoop framework, a distributed file system that can conveniently run on commodity hardware for processing unstructured data. The dataset is stored as Resilient Distributed Dataset(RDD), which shows great performance in processing big data. The output are the extracted interval sequence and duration sequence.

The specific implementation of the algorithm is showed in algorithm 1. First, in line 1–2, each record of CNEPD is changed to {*Segment*, (*Date*, *IP*, *Segment*, *Username*, *Count*)}. Key-Value pairs are grouped according to *Segment*, the key. Then, in line 3–18, *Date* of each group is extracted and sorted in ascending order to form a probing time sequence. Afterwards, time sequences of duration and interval are respectively calculated in line 6–7 and 9–18. Finally, the set of time characteristics is obtained.

**Algorithm 1:**
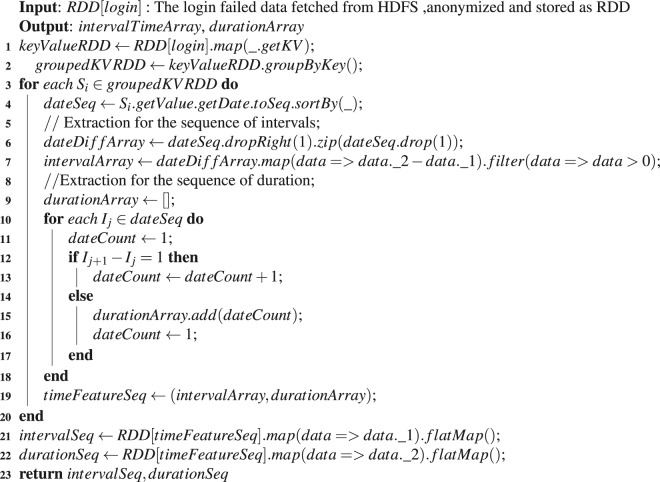
Date Feature Extraction Algorithm.

### Construction of network model

**Figure 2:**
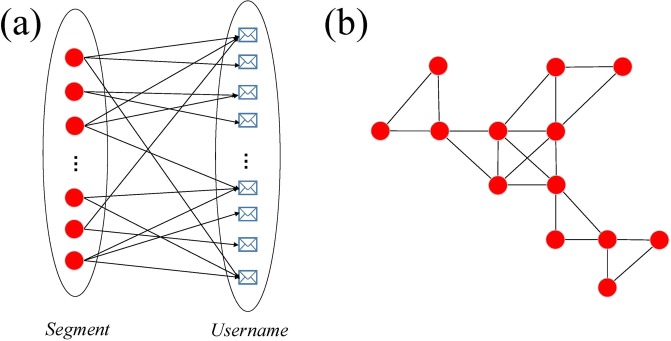
Network Model Construction. (**a**) The attack association bipartite graph shows the relationship between network segments and usernames.The red nodes and the envelope-shaped nodes represent attackers and e-mail accounts,respectively. Each line represents a probing attack. (**b**) The probing source mapping network is formed from (**a**) by one mode projection.

One of important probing characteristics is the choice tendency of targets. From CNEPD, we can get a list of probed e-mail accounts. If we desire to figure out attackers choice tendency, we need to understand the relationship between probing sources and probed accounts from our dataset. Hence, we construct a network based on the fields of “*Segment*” and “*Username*”. In this case, an attack association bipartite graph, the probing relationship network, is constructed based on two fields of CNEPD, as shown in Fig. 2(a).

From CNEPD, we can conclude that there are a large number of attackers probe different accounts in the whole time. If we desire to understand the probing pattern of attackers, it is important to figure out if there is a relationship between the attackers. In order to analyse the correlation between attackers, according to^22^, we change the probing relationship network into the probing source mapping network, which is composed of attack nodes by one mode projection. As shown in Fig. 2(b), each node represents an attack network segment, and an edge between two nodes indicates that both segments have probed the same one or more e-mail accounts. By constructing two network models, the relationship between segments and usernames as well as the relationship among attackers can be analysed.

### Method of node distribution attribute analysis

In a network system, the distribution attribute of nodes is one of the most important characteristics to describe the network. In order to mine the important nodes and find out if there is any relationship between nodes, we analyse the distribution of nodes in the 2 network models. The degree of node is the most basic attribute of node’s distribution, which represents the number of edges connected with the node. The degree of node *i* can be expressed as *k*_*i*_. In scale-free networks, the distribution of nodes’ degree usually fits power law distribution. The distribution of nodes degree can be expressed as:

1 $${p}_{k}\sim {k}^{-\gamma }.$$

In the probing relationship network and the probing source mapping network, the more important a node is, the more likely it is to have similarity and relevance with other nodes in probing pattern. In order to figure out the distribution of probing nodes and the relationship between them in the network, we describe the importance of nodes by calculating the centrality of nodes based on nodes degree. In our work, we focus the following four types of centrality.

1. ***Degree Centrality*** is the degree of node, which refers to the number of edges of a node. The degree centrality of node *i* is defined as:
  2 $${k}_{i}=\mathop{\sum }\limits_{j=1}^{n}\,{a}_{ij}.$$
2. ***Closeness Centrality*** is defined as the reciprocal of the average distance from one node to the other nodes in the network. The closer the average distance from one node to the other, the more central the node is. The closeness centrality of node *i* is defined as:
  3 $${C}_{C}(i)=\frac{n-1}{{\sum }_{j}{d}_{ij}}.$$
3. ***Betweenness Centrality*** describes the path of node information transmission. Nodes with high betweenness centrality are the nodes that transmit the most information. In the probing source mapping network, if one nodes betweenness centrality is high, it means that this node may have the most similar probing behaviour with other nodes. The betweenness centrality of node *i* is defined as:
  4 $${C}_{B}(i)=\sum _{st}\,\frac{{n}_{st}(i)}{{g}_{st}}.$$
  From Eq. (4), *st* is a pair of nodes in the network, *g*_*st*_ is the total number of shortest paths from *s* to *t*, and *n*_*st*_(*i*) is the number of nodes in one shortest path.
4. ***Eigenvector Centrality*** is an extension of degree centrality. It increases with the increase of the importance of one nodes neighbour nodes. The eigenvector centrality of a node is proportional to the sum of eigenvector centrality of the node s neighbour nodes. The higher eigenvector centrality of one node is, the more important its neighbour nodes are, which indicates that the node is very important in the network. The eigenvector centrality of node *i* can be define as:

5 $${x}_{i}={k}_{l}^{-1}\sum _{j}\,{A}_{ij}{x}_{j}.$$

From Eq. (5), *k*_*i*_ is a constant and *A*_*ij*_ represents the adjacency matrix of the network.

**Figure 3:**
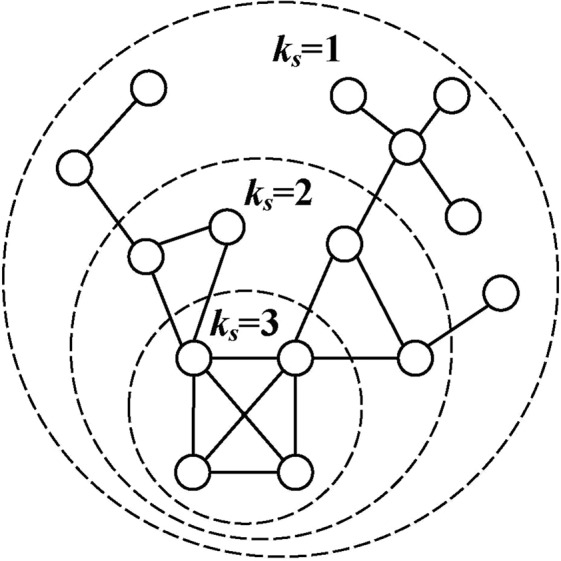
A simple example of k-core. Dotted lines divide the network into three layers.

In addition to the above 4 types of centrality, we also use the K-shell algorithm^23^ to measure the importance and distribution of nodes in the network. K-shell, also known as k-core, divides the network into layers from the centre to the periphery. The K-shell value of a node is marked as *k*_*s*_. In this algorithm, the nodes with degree 1 from the network are firstly deleted. At the same time, new nodes with degree 1 may appear in the network. Then continue to delete them until there are no nodes with degree 1 in the network. All the deleted nodes *k*_*s*_ value is 1. These nodes constitute the shell of *k*_*s*_ = 1. Next, nodes with degrees of 2, 3, 4 to the maximum *n* are deleted in the same way. After that, the network is divided into *n* shell layers. All nodes in the same layer has the same *k*_*s*_ value. Figure 3 demonstrates a simple example of k-core structure.

Beside individual node attributes, the discretization and aggregation of nodes are also important to reflect the characteristics of network structure.Clustering coefficient describes the degree of clustering of nodes in the network, which reflects the tendency of nodes with neighbours in common to be directly connected^24^. For the probing source mapping network, clustering coefficient reflects the possibility of direct association between attackers. Local clustering coefficient is used in this paper, which represents the probability that any pair of neighbour nodes of one node are directly adjacent to each other. Local clustering coefficient *c*_*i*_ is defined as:

6 $${c}_{i}=\frac{2{E}_{i}}{{k}_{i}({k}_{i}-1)}.$$

From Eq. (6), *E*_*s*_ is the number of node *i* s neighbour nodes which are directly adjacent. $\frac{{k}_{i}({k}_{i}-1)}{2}$ is the number of possible neighbour pairs of node *i*.

## Results and Analysis

### Results and analysis of time feature

**Figure 4:**
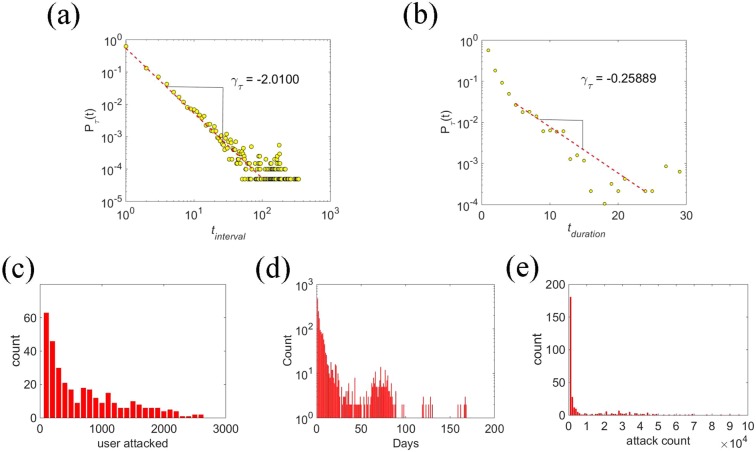
Distributions of different time features. (**a**) Probability distribution of probing intervals.The red line is the best fit to the power-law distribution, which gives the power-law exponents *γ* = −2.0100. (**b**) Probability distribution of probing duration.The red line is the best fit to the exponential distribution, which gives the exponents *γ* = −0.25889. (**c**) The distribution of the number of days over the number of probed accounts per day. (**d**) The distribution of the number of network segments over the total number of days per segment attack. (**e**) The distribution of the number of network segments over the number of attacks per network segment per day.

The probing interval and duration of CNEPD are calculated according to algorithm 1. The probing interval and duration sequences are obtained and the distribution of these two features are calculated respectively. Then the distribution fitting curve is calculated by the maximum likelihood estimation method, and the results are shown in Fig. 4(a,b).

Figure 4(a) demonstrates the distribution of probing intervals, which follow a power-law distribution. The red line corresponds to the power-law fits with exponents *γ* = −2.01. Most probing intervals are short, which are less than ten days. There are a few outliers with long intervals in the tail because there are probing intervals of some network segments which are longer than 100 days. The result indicates that attackers prefer to make persistent probes at short intervals.

Figure 4(b) shows the distribution of probing duration. It fits the exponential distribution with the slope of −0.25889, which manifests the memoryless property. The probing duration of each network segment is less than 30 days, and most of the continuous attacks have a short duration. From the results, we can get the conclusion that attackers prefer probing with short duration.

The average number of probing attacks per day in CNEPD is close to 1000. According to daily probing attacks, numbers of probed accounts per day are calculated and the result is shown in Fig. 4(c). From Fig. 4(c), it can be seen that about a fifth of the time, the number of daily probed accounts ranges from 0 to 100. Furthermore, the number of probed accounts in four fifths of the time exceeds 100, the maximum number even reaches 2600. It can be seen from the above results that the numbers of probe attack and probed accounts every day are both quite large.

In order to analyse the attack behaviour of each single probing source, we calculate the distribution of the number of probing attacks. The distribution for each probing source and the distribution for each account are shown in Fig. 4(d,e).

Figure 4(d) demonstrates that most of the network segments appear within 100 days, and the number of attackers probing less than 30 days account for the most. Figure 4(e) shows that most of the attack counts are concentrated in the range of abscissa value <10000. From the Fig. 4(d,e), we can find that most of the network segments probe e-mail accounts in few days, and they only generate several attacks on probing days. The results indicate that the actual probing frequency of each network segment is low, and the number of probing in these 333 days is small as well.

From the above results, we can come to a conclusion that the main characteristics of probing are short duration and intervals. As for the number of probing attacks and robed accounts, both of them are fairly large. However, from the point of view of each single node, nodes’ behaviours have the characteristics of low frequency and small amount of attacks in time distribution. Attackers’ probing pattern shows strong concealment because of low frequency but harmful impact as a result of the huge number of attacks in the entire data.

### Results and analysis of the probing relationship network

**Figure 5:**
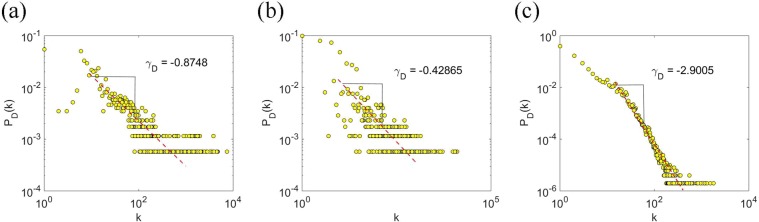
Node degree distribution of the probing relationship network. (**a**) Probability distribution of attack nodes’ degrees.The red line is the best fit to the power-law distribution, which gives the power-law exponents *γ* = −0.8748. (**b**) Probability distribution of attack nodes’ weights.The red line is the best fit to the power-law distribution, which gives the power-law exponents *γ* = −0.42865. (**c**) Probability distribution of edges’ weights.The red line is the best fit to the power-law distribution, which gives the power-law exponents *γ* = −2.9005.

We construct the probing relationship network based on CNEPD. According to the aggregation of IP segments, the degree distribution of nodes in the network is calculated. The results are shown in Fig. 5(a). The degree distribution of attack nodes fits power-law distribution with the exponent *γ* = −0.8748. Degrees of most nodes are less than 10, which means the majority of network segments probe no more than 10 accounts in the whole time. Besides, attackers only probe a few accounts, and the proportion of probing sources which attack a large number of accounts in the whole dataset is low as well. The results demonstrate that the number of accounts probed by most probing sources is quite little.

In the probing relationship network, each edge between two nodes has a weight, which indicates the number of attacks from the attacker to the target. We calculate the number of attacks launched by each network segment and get the distribution of the sum of edges’ weight per probing node. The results are shown in Fig. 5(b). The weight distribution of attack nodes fits power-law distribution with exponent *γ* = −0.42865 and weights of most nodes are less than 100. The result shows that most segments’ only probe a few times and there are only few nodes with larger weights. Attackers like to use multiple network segments rather than single segment or IP address to carry out probes

We take “segment-username” as a connection, aggregating the same connection, calculating the number of attacks. The distribution of connection weights is shown by Fig. 5(c). The distribution of the connection weights fits power-law distribution,with exponent *γ* = −2.9005. The result indicates that when network segments probe each account, they only probe a few times.

From the above analysis, we find that the number of accounts probed by attackers and attackers only probe a few times for each account, although the number of probing attacks in the whole dataset is more than 3,000,000. Many network segments each generate no more than 100 probing attacks in these 333 days. Generally speaking, a large number of probing sources are each aiming at a few mount of accounts. These segments only launch several attacks for each account.

### Results and analysis of the probing source mapping network

**Figure 6:**
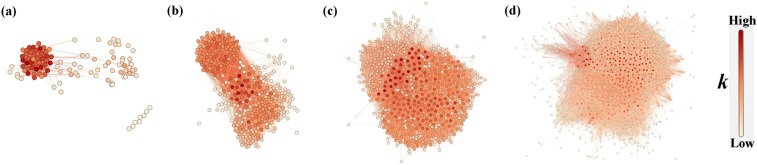
Examples of the probing source mapping network constructed by different size of data: (**a**) Attack data in 1 day; (**b**) Attack data 1 week; (**c**) Attack data in 1 month; (**d**) The entire data set. Colour of nodes deepens with the value of their degree *k*.

In the probing source mapping network, each node represents a network segment, a probing source. An edge between two nodes indicates that the two nodes have probed the same one or more e-mail accounts. In order that we can understand the relationship between attackers roughly, as shown in Fig. 6, there are examples of the network constructed based on the data of one day, one week, one month and the entire data set. With the accumulation of time, the network becomes increasingly complex, the number of nodes and edges increases greatly. CNEPD totals 1737 network segment nodes and 88,040 edges. Because of complex connections among nodes, we initially deduce that there are strong correlations among a large number of nodes, which might have similar attack behaviours. The next content will analyse the network construction and characteristics of nodes to describe attack behaviours.

#### Analysis of the probing source mapping network construction

**Figure 7:**
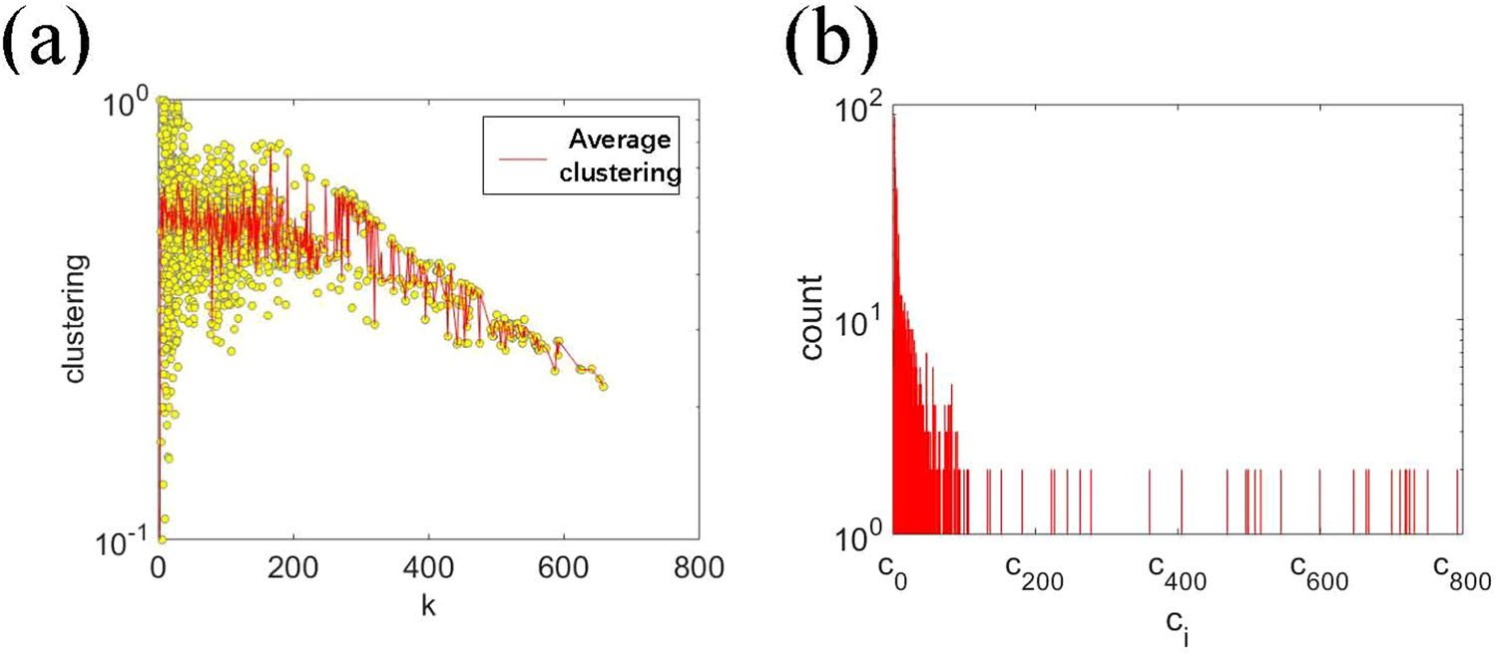
Characteristics of the probing source mapping network. (**a**) The distribution of the clustering coefficient over the degree of nodes.The red line stands for the average value of clustering coefficient. (**b**) The distribution of the number of complete sub-graphs in the network.

After a preliminary analysis of the probing source mapping network, we conclude that there is a high correlation between the nodes. In order to analyse the network construction and figure out the relationship between attackers, we calculate the degree and clustering coefficient of each node. The result is shown in Fig. 7(a). From the result, we can see that degrees of nodes fluctuate greatly with the clustering coefficient. However, the average clustering coefficient decreases slightly with the increase of degree. Beside that, nodes with higher degree are with bigger average clustering coefficient as well. The result indicates that, in the network, there are many clustering groups of nodes which contain a huge amount of large-degree nodes. In other words, probing behaviours of these nodes are intensely similar, aiming at the same one or several e-mail accounts.

To confirm the similarity of different nodes’ probing behaviours, we calculate the characteristics of complete sub-graphs in the network. For most networks, a complete sub-graph represents that all nodes in it are strongly correlated. In the probing source mapping network, each complete sub-graph indicates that all nodes in it have probed the same one or several e-mail accounts. The number of complete sub-graphs is calculated according to the constructed network, as shown in Fig. 7(b). It can be seen that the distribution of complete sub-graphs is dispersed. Although the number of nodes in most complete sub-graphs is less than 100, there are still large complete sub-graphs. The largest complete sub-graph contains more than 800 nodes, which indicates that more than 800 segments have probed the same one or several e-mail accounts.

From the above results, there are multiple network segments probing the same one or more e-mail accounts. Considering the conclusion of last subsection that single network segment has fewer times to probe an account, the result explains why the average number of attacks for each e-mail account is large, while the actual number of attacks of each network segment is small. Although each segment only probes a few times, multiple attackers probe accounts in a cooperative way. These probing nodes actually generate a huge number of attacks, posing a threat to e-mail accounts.

#### Analysis of the node characteristic

**Figure 8:**
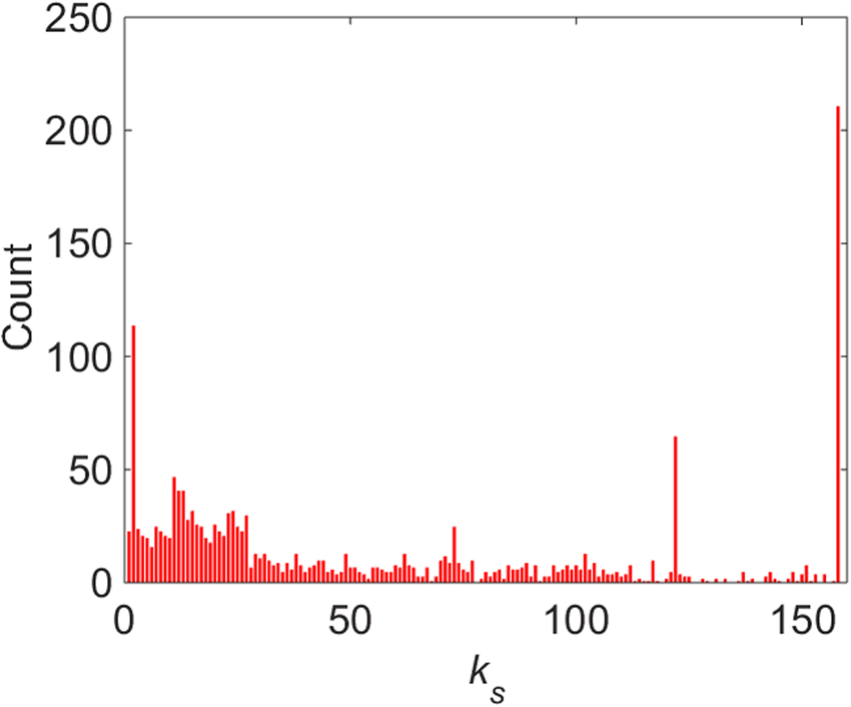
The distribution of the number of nodes over different *k*_*s*_ value.

In order to figure out the distribution of nodes in the probing source mapping network, we decompose the network by k-shell and the result is shown in Fig. 8. The distribution of *k*_*s*_ is quite different, and many of nodes are located in the most exterior and interior. A large number of external nodes (located in the outer shell of *k*_*s*_ < 10) are free in the outer layer of the network with a few attacks.The maximum *k*_*s*_ is 158, and the number of nodes with *k*_*s*_ = 158 is 211.

In some networks, the core nodes are the most influential and important. However, nodes in high shells, the innermost core, are not good spreaders with much influence in other networks^25^. In order to verify that these core nodes are actually important in the network, we calculate the centrality of all nodes and then rank them. The results are shown in Table 2.

**Table 2:** Top 20 probing node IDs of 4 types of centrality.

Ranks	Degree Centrality	Closeness Centrality	Betweenness Centrality	Eigenvector Centrality
1	694*	694*	957*	785*
2	994*	785*	694*	994*
3	785*	994*	785*	201*
4	880*	880*	880*	305*
5	777*	777*	994*	694*
6	201*	957*	777*	880*
7	711*	201*	711*	777*
8	305*	711*	264*	1308*
9	957*	305*	440*	711*
10	1181*	1181*	333*	1073*
11	1219*	1219*	988*	1181*
12	1441*	1441*	1442*	171*
13	618*	618*	1219*	1219*
14	1073*	1073*	890*	1616*
15	757*	757*	329*	961*
16	1602*	1602*	1400*	1518*
17	961*	961*	535*	326*
18	326*	326*	996*	1624*
19	171*	171*	1181*	58*
20	1477*	1477*	825*	791*

Nodes in the core shell are marked with *.

As a result, all the nodes in Table 2 locate in the shell of *k*_*s*_ = 158, which imply that there is a great correlation and similarity between these core nodes in their probing behaviours. These nodes are with a large degree and high centrality. We can speculate that there is a cooperative probing behaviour on some e-mail accounts by a part of probing sources together.

From the above results, it can be found that from the view of the entire data set, probing sources of large-degree nodes are highly correlated with many shared edges. Besides, the number of nodes in the largest sub-graph is quite large as well, and there is a overlap in the probing targets of different attack nodes. In other words, it manifests that there is a strong cooperation between probing sources in CNEPD. These nodes attack a batch of accounts in a collaborative way for a long time. According to results, we speculate that these probed accounts may be easily accessible public e-mail ones (such as some accounts with contact information disclosed on public websites of campus), which are of great value to attackers behind these probing sources.

### Conclusion of the probing attack

Through the above analysis results, we can define this kind of attack as the Distributed E-mail Cooperative Probing Attack. Characteristics of the attack are as follows: (1) It possesses a distributed probing pattern and the number of attack sources is quite large. (2) It attacks a lot and the frequency is high, both the number of probing attacks and the number of probed accounts in the whole time are large, and the distribution of probing time is relatively dense. (3) Each single node probes few and the attack frequency is low, each single node probe few accounts and the number of probing attacks is small as well. (4) With highly cooperative probing sources, with high correlation between probing sources, there is a certain overlap in targeted accounts, demonstrating strong cooperation.

This kind of probing pattern possesses characteristics of well-covertness, difficulties of detection and harmful effects. As for a single probing source, because of its low frequency and few probing times, it can effectively reduce the detection rate of traditional security equipment. In addition, from the entire dataset point of view, this kind of attack produces a huge number of probes, and its effects are no less than probing a large number of accounts in a short period of time, such as database collision and violent cracking. Furthermore, probing sources are highly correlated. In a way of cooperation, the behaviour of continuous probing attacks has brought great security risks for accounts repeatedly probed by attackers.

## Discussions

In this paper, we define a kind of distributed e-mail cooperative attack, and analyse its probing pattern. Results of analysis in our work can enable security administrators to propose targeted detection strategies based on the its behaviour characteristics. At the same time, analysis results have been submitted to the security departments of campus network, reminding e-mail users to strengthen protection of their accounts and privacy security to reduce the risk of asset losses. It is worth mentioning that we analyse the attack pattern based on CNEPD, the dataset collected in campus network. Then we make full use of the characteristics in dimensions of time and correlation space, analysing its characteristics and depicting the attack pattern. In addition, as far as we know, methods of graph mining applied to the analysis of e-mail login data are innovative. Besides, this paper can also provide some help for methods of graph mining in analysing security data, which has certain reference significance.

According to the characteristics of the probing pattern, the following strategies can be adopted for detection. (1) Increase the time window of detection program. Keep as much data as possible to analyse, so as to find out the accumulated probing records from a long time span. (2) Expand the range of detection targets. Targets of detection and analysis should not be limited to a single IP address or class C network segment. Beyond that, relevant thresholds and baselines should be set by analysing the entire data, so as to improve the detection rate of such distributed and cooperative probing sources.

As future works, we plan to focus on the dynamic evolution trend of CNEPD, study the development law of probing behaviours in time dimension, and realize the prediction of such probing attacks. On the other hand, based on the characteristics we propose in this paper, we can implement a detection system for such attacks.

## Data Availability

Our data set is available http://csri.scu.edu.cn/news/728. The data set is divided into eight parts due to the size of uploaded files required by the website.
